# *Cryptococcus neoformans* Capsular GXM Conformation and Epitope Presentation: A Molecular Modelling Study

**DOI:** 10.3390/molecules25112651

**Published:** 2020-06-07

**Authors:** Michelle M. Kuttel, Arturo Casadevall, Stefan Oscarson

**Affiliations:** 1 Department of Computer Science, University of Cape Town, Cape Town 7701, South Africa; 2 Department of Molecular Microbiology and Immunology, Johns Hopkins Bloomberg School of Public Health, Johns Hopkins University, 615 N Wolfe St Room E5132, Baltimore, MD 21205, USA; acasade1@jhu.edu; 3 Centre for Synthesis and Chemical Biology, University College Dublin, Belfield, Dublin 4, Ireland; stefan.oscarson@ucd.ie

**Keywords:** *Cryptococcus neoformans*, conjugate vaccines, GXM, conformation, capsular polysaccharide, epitope, carbohydrate antigen, molecular modelling

## Abstract

The pathogenic encapsulated *Cryptococcus neoformans* fungus causes serious disease in immunosuppressed hosts. The capsule, a key virulence factor, consists primarily of the glucuronoxylomannan polysaccharide (GXM) that varies in composition according to serotype. While GXM is a potential vaccine target, vaccine development has been confounded by the existence of epitopes that elicit non-protective antibodies. Although there is evidence for protective antibodies binding conformational epitopes, the secondary structure of GXM remains an unsolved problem. Here an array of molecular dynamics simulations reveal that the GXM mannan backbone is consistently extended and relatively inflexible in both *C. neoformans* serotypes A and D. Backbone substitution does not alter the secondary structure, but rather adds structural motifs: βDGlcA and βDXyl side chains decorate the mannan backbone in two hydrophillic fringes, with mannose-6-O-acetylation forming a hydrophobic ridge between them. This work provides mechanistic rationales for clinical observations—the importance of O-acetylation for antibody binding; the lack of binding of protective antibodies to short GXM fragments; the existence of epitopes that elicit non-protective antibodies; and the self-aggregation of GXM chains—indicating that molecular modelling can play a role in the rational design of conjugate vaccines.

## 1. Introduction

*Cryptococcus neoformans* is an opportunistic invasive fungus that causes serious disease (typically meningitis) in immunosuppressed hosts [1]. *Cryptococcus* species are encapsulated fungi, with a thick, hydrophillic capsule that covers the cell wall surface, forms a protective biofilm and is the chief virulence factor [2]. The capsule is primarily composed of a glucuronoxylomannan polysaccharide (GXM, around 90%); further minor components are a galactomannan polysaccharide (GXMGal, 10%) and mannoproteins (<1%) [3]. *C. neoformans* is currently classified into two varieties known as *neoformans* and *grubii* [4]. There are three serotypes of *C. neoformans*, referred to as serotype D (*Cryptococcus neoformans* var. *neoformans*), serotype A (*Cryptococcus neoformans* var. *grubii*) and serotype AD (hybrid). Globally, serotypes A and D are responsible for the vast majority of infections in humans [4]. Serotypes B and C are now classified as a separate species, *Cryptococcus gattii*. However, genomic analysis suggests that both the *C. neoformans* and *C. gattii* groupings include various species and these are currently viewed as species complexes [5].

**Figure 1:**
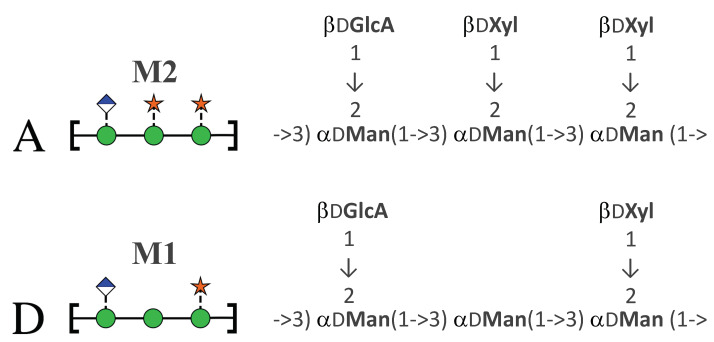
The predominant GXM repeat unit motif (triad) in *C. neoformans* serotypes. Serotype A (*Cryptococcus neoformans* var. *grubii*, top) has predominantly the M2 triad and serotype D (*Cryptococcus neoformans* var. *neoformans*, bottom) the M1 triad [11]. The RUs are shown on the left with the SNFG symbols [13,14].

The serotype classifications for *Cryptococcus* are on the basis of antigenic differences arising from structural variations in GXM [6]. Light scattering and hydronamic studies suggest that GXM is a branched polymer comprising an α(1→3) mannose backbone substituted with β(→2) glucuronic acid every third mannose, along with variable β(1→2) and β(1→4) xylose side chains [7,8]. Some mannose residues are 6-*O*-acetylated (up to 60%) and O-acetylation plays a key role immune recognition of GXM [9]. The GXM polysaccharide has considerable heterogeneity [10]: in contrast to bacterial polysaccharides that have a single oligosaccharide repeat unit (RU), GXM has at least six repeat motifs (called triads), and a single molecule of GXM may include different motif combinations [11]. For each *Cryptococcus* serotype, the GXM structure has a major triad and one or more minor triads. For serotype A, the dominant triad is a six-residue RU with two β(1→2) xylose side chains (triad M2), whereas serotype D has a dominant five-residue RU with one β(1→2) xylose side chain (triad M1), as shown in Figure 1. Serotypes A and D have an estimated average of two acetates per triad [12].

GXM is a promising antigen in vaccine development since a polysaccharide-protein conjugate vaccine could potentially provide effective protection [15]. However, there have been complications following this route, because the GXM polysaccharide is poorly immunogenic and has been found to elicit both protective and non-protective and even deleterious antibodies in humans [16]. This led to the hypothesis that GXM contains some epitopes eliciting protective antibodies and other epitopes eliciting non-protective antibodies, with a non-protective antibody response impeding the formation of protective antibodies [17]. There is evidence that some epitopes that elicit protective antibodies are conformational [18] and O-acetylation is known to be critical for binding of protective antibodies [19,20]. One approach to identifying protective GXM epitopes is the creation of a library of synthetic oligosaccharides which are then conjugated to protein carriers and tested in mice to identify immunogenic motifs that are potential vaccine candidates [18,21]. In the first stages of this strategy, none of the smaller synthesised molecules (from a tetra to a heptasaccharide) were recognised by protective antibodies; a synthetic heptasaccharide representing the GXM serogroup A triad (M2) conjugated to human serum album raised a non-protective immune response in mice [17]. However, recently a synthetic decasaccharide was shown to bind to a number of protective antibodies and is therefore a promising vaccine candidate [18].

It has been hypothesised that differences in the degree of side-chain substitution and O-acetylation give rise to changes in the secondary structure of the GXM polysaccharide that directly affect Ab binding and hence antigenicity [9]. However, despite postulates of the existence of conformational epitopes of GXM [9,11,17], the secondary structure of GXM is still unknown and is recognised as a gap in the understanding of *C. neoformans* capsule [20]. In addition, the mechanisms through which the GXM polysaccharides (and other capsule molecules) assemble into a capsule remain largely undiscovered [20], although there are indications that GXM molecules self-aggregate, possibly mediated by divalent cations [22,23,24]. In the absence of experimental evidence on secondary structure (which is extremely challenging to obtain for flexible polysaccharides), molecular modelling has been demonstrated to provide insights into molecular conformation, biophysical dynamics and interactions that can usefully inform vaccine development [25].

**Figure 2:**
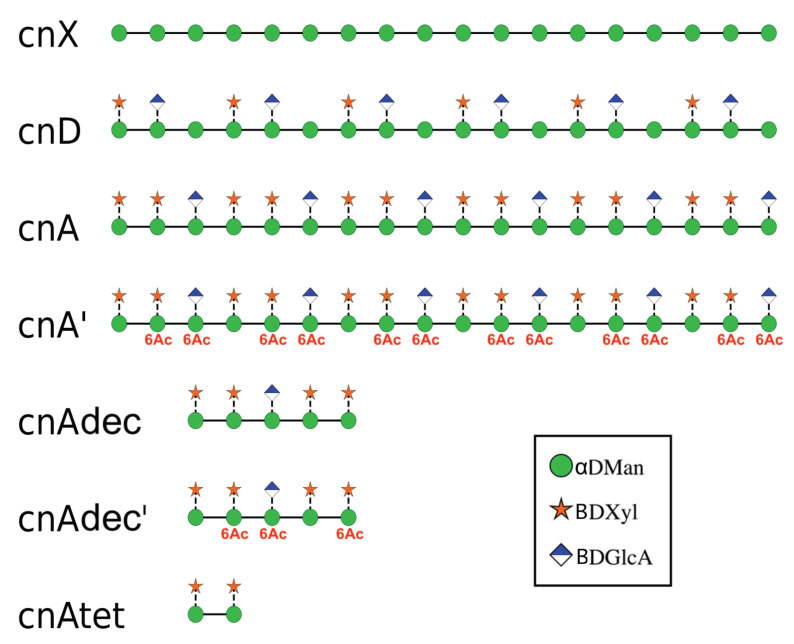
The array of GXM oligosaccharides simulated in this work, shown with the SNFG symbols [13,14] for the sugar residues. Six RUs of an unsubstituted backbone (cnX); the main repeat motifs in serogroup D (cnD) serogroup A (cnA) and 6-*O*-acetylated serogroup A (cnA’) were run in separate simulations; and three shorter strands of serogroup A GXM: a decasaccharide (cnAdec), a 6-*O*-acetylated decasaccharide (cnAdec’) and a tetrasaccharide (cnAtet).

In this work we employ molecular dynamics simulations on an array of oligosaccharides (Figure 2) to establish the conformation of GXM in serotype A and D, aiming to investigate the following questions.

1. What is the secondary structure or conformation of GXM?
2. Does the differing xylose substitution pattern in serotypes A and D alter the GXM conformation?
3. Does 6-O-acetylation on the mannose backbone alter the conformation of serotype A?
4. Do shorter strands with the M2 triad have the same conformational epitope as the GXM serotype A polysaccharide?
5. What is mechanism of GXM self-aggregation?

To investigate the effects of the side chain substitutions on GXM conformation, we ran molecular dynamics simulations in an aqueous solution of 6 RUs of an unsubstituted α(1→3) mannose backbone (hereafter termed cnX), for comparison purposes, and simplified 6-RU chains of serogroup A (cnA) and serogroup D (cnD). For the simplified strands, the saccharide chains comprised simple repeats of the dominant triad motif for the serogroup (as shown in Figure 2). To investigate the effect of O-acetylation on GXM, we simulated serogroup A with 6-O-acetylation on the first and third mannose in the RU (cnA’). This pattern was chosen to match that in the synthetic decasaccharide [18] and is in accordance with the estimated average of two acetates per triad [12]. Then, to investigate the effect of chain length on conformation, we simulated a tetrasaccharide (cnAtet) as well as the decasaccharide representing promising vaccine candidate, the latter both being 6-*O*-acetylated (cnAdec’) and de-O-acetylated (cnAdec). Finally, to look at the possibility of GXM self-aggregation, we ran a simulation of cnA and cnD together, to allow for possible spontaneous association of the chains. The trajectories of these multiple simulations were then analysed and compared, as discussed below.

## 2. Results

We first compare the effects of substitution of the GXM mannan backbone on chain flexibility and proceed to a comparison of the secondary structure in cnD, cnA and cnA’. We then contrast the conformation of shorter fragments of serotype A with the 6-RU strands. Finally, we look at the potential for GXM chain self-aggregation.

### 2.1. Comparison of GXM Chain Flexibility

**Figure 3:**
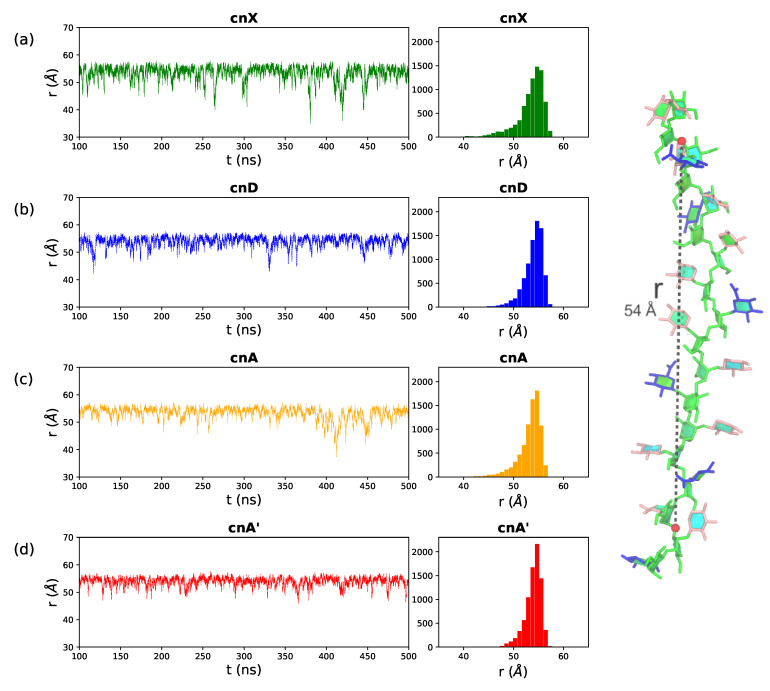
End-to-end distance, *r*, time series and corresponding histograms for the (**a**) cnX, (**b**) cnD, (**c**) cnA and (**d**) cnA’ GXM chains. The first 100 ns of simulation are considered equilibration and are not shown. Here *r* is defined to exclude the two terminal residues on either end of the chain, as the distance from O3 in the second linkage to O3 in the 16th linkage in the 18-mannose backbone—labelled for cnA in the image on the right. The residues and substitutions are coloured as follows: αDMan—green; βDGlcA—blue; βDXyl—pink; and 6-OAc—red.

A simple measure of chain flexibility is the fluctuation in end-to-end distance, *r*, for a molecule over the course of a simulation. Figure 3 shows the *r* time series and corresponding histograms for (a) cnX, (b) cnD, (c) cnA and (d) cnA’. Here we define *r* for all 6-RU GXM chains as the distance from O3 in the second linkage in the mannose chain to the O3 in the second last linkage, thereby excluding the more flexible two terminal residues on either end of the chain (labeled on the cnA molecule in Figure 3, right). The α(1→3) mannan chain is generally extended: the distributions of *r* (Figure 3 right column) are tight and skewed to the right (larger *r* values), with all the four saccharides having a narrow peak at the median chain length of 54 Å. The mannan backbone is thus remarkably inflexible, as there are no short *r* distances indicating bends that bring the ends of the chain into close proximity; although transient “elbow” bends do occur occasionally, they do not persist. Further, comparison of the *r* plots shows a trend of decreasing chain flexibility with increasing chain substitution: the unsubstituted cnX backbone is the most flexible with the broadest spread in *r* (σ = 2.8); this flexibility is decreased in cnA (σ = 2.3) and the most substituted cnA’ is markedly the least flexible, with the narrowest range of *r* (σ = 1.6).

**Figure 4:**
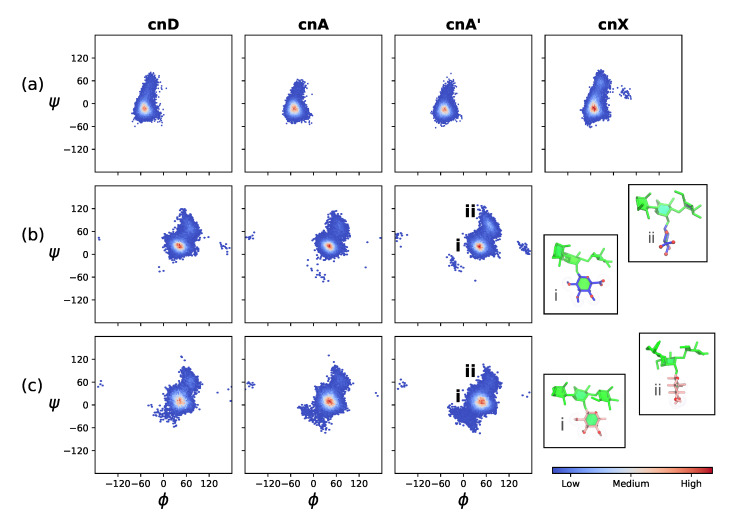
Heat maps of the glycosidic linkage orientation distribution over the last 400 ns of simulation in the two central RUs (RU3 and RU4) in the GXM chains. Row (**a**) shows the backbone αDMan(1→3)αDMan linkage, row (**b**) the βDGlcA(1→2)αDMan side chain linkage and row (**c**) the βDXyl(1→2)αDMan side chain linkage. Glycosidic linkage orientations are shown as rotations of the linkage dihedral angles ϕ and ψ. The insets in row (**b**,**c**) show the major face-on (**i**) and minor edge-on (**ii**) orientations of the β(1→2)-linked side chains relative to the mannan backbone. The residues and substitutions are coloured as follows: αDMan—green; βDGlcA—blue; βDXyl—pink; and 6-OAc—red.

This trend of reduced flexibility with increased backbone substitution is further illustrated by the decreasing range of rotation in the backbone mannose α(1→3) linkages, shown in the heat maps in Figure 4a. Although the dominant orientation of this linkage is the same in cnX, cnD, cnA and cnA’ (indicating the same backbone conformation in all 6-RU GXM molecules), the range of motion is greatest in cnX and decreases in the order cnD, cnA and cnA’.

**Figure A1:**
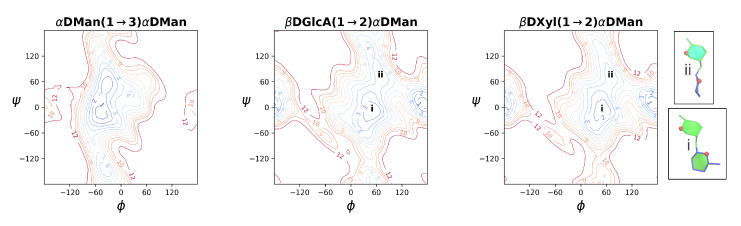
Contoured 2D ϕ, ψ PMF surfaces for disaccharide in the gas phase illustrate the range of motion possible for an unrestrained linkage: αDMan(1→3)αDMan (**left**); βDGlc(1→2)αDMan (**middle**); and βDXyl(1→2)αDMan (**right**). Contours are drawn at intervals of 1 kcal.mol^−1^ to a maximum of 12 kcal.mol^−1^. The β(1→2) PMF maps are annotated with the ϕ, ψ regions associated with the (i) major “face-on” and (ii) minor “side-on” linkage orientations. The insets show schematics of the relative rotations of the glycosidic rings in these two orientations.

The orientation of the βDGlcA(1→2)αDMan (Figure 4b) and βDXyl(1→2)αDMan (Figure 4c) side chains does not vary greatly across cnD, cnA and cnA’. However, the side chains are considerably more mobile than the backbone (compare the narrower extent of the heat maps in Figure 4a). Both side chains are predominantly in a “face-on” orientation to the mannan backbone (insets i in Figure 4b,c), where the plane of the ring and hence hydrophillic ring hydroxyls are aligned parallel to the mannan backbone. This orientation is the global energy minimum for the βDGlcA(1→2)αDMan on the vacuum PMF (Appendix A, Figure A1). The side chains also have a minor "edge-on" conformation (insets ii in Figure 4b,c), where the plane of the ring and hence hydrophillic ring hydroxyls are aligned perpendicular to the mannan backbone. This orientation is a local energy minimum for the βDGlcA(1→2)αDMan on the vacuum PMF (roughly 2 kcal.mol^−1^ above the global minimum, Figure A1). Note that a comparison of cnA and cnA’ in Figure 4 shows that 6-O-Ac substitution does not significantly affect orientation of the βDGlcA and βDXyl side chains.

### 2.2. Comparison of GXM Chain Conformations and Binding Surfaces

**Figure 5:**
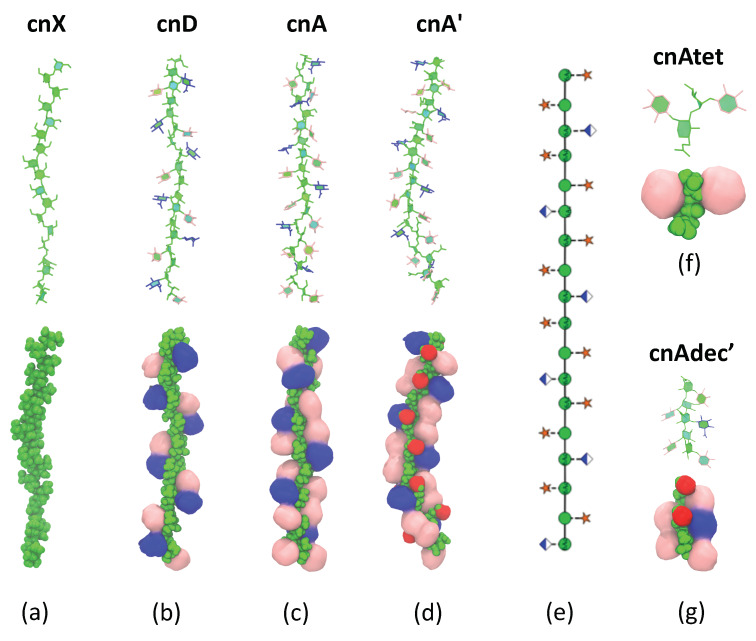
Dominant conformations of the GXM molecules shown with two representations in the VDM package [26]—the PaperChain visualisation [27] to highlight the conformation of the backbone and space-filling representations to highlight the exposed binding surface. Representative conformations are shown for (**a**) the unsubstituted mannan backbone cnX; (**b**) cnD; (**c**) cnA; (**d**) and cnA’ (6-*O*-acetylated). A schematic for the arrangement of the side chain substitutions is shown in (**e**) using the SNFG symbols for the sugar residues [13,14]. Representative conformations of the chain fragments are shown for the tetrasaccahride (**f**) cnAtet and the 6-*O*-acetylated decasaccharide (**g**) cnAdec’. The chain substitutions in all representations are coloured as follows: αDMan—green; βDGlcA—blue; βDXyl—pink and 6-OAc—red.

Although the flexibility of the GMX molecules decreases with increasing substitution, the backbone conformation does not change significantly. Conformational clustering reveals that all the GXM chains have a strongly dominant molecular conformation, shown for each of the GXM molecules in Figure 5. For all molecules, this dominant conformation corresponds to the peak in the *r* histogram (see Figure A3). In addition, the prevalence of this primary conformation increases in the order cnX (51%) < cnD (55%) < cnA (57%) < cnA’ (69%)—A further indication that in GXM the chain flexibility decreases with increasing chain substitution. In all conformations, the backbone is extended, as is most apparent in the unsubstituted cnX conformation (Figure 5a), but is also clear for cnD (Figure 5b), cnA (Figure 5c) and cnA’ (Figure 5d). The backbone twists dynamically from flatter ribbon-like conformations to extended helical conformations, but its behaviour is relatively unaffected by the presence or absence of side-chain substitutions or O-acetylation. The sole effect of increasing substitution on the backbone is to constrain rotation about the backbone linkages. Therefore, the theory that the conformation of the backbone is changed with altered substitution patterns [9] is not supported by our results.

The βDGlcA and βDXyl side chains are arranged along the backbone in two hydrophillic fringes, primarily in the “face-on” orientation with the ring hydroxyl groups aligned in the same plane as the mannan backbone. Side chains in the same fringe are in close proximity to each other and interact with each other via a dynamic network of hydrogen bonds, shifting closer and further away within a restricted range of 10 Å (see Appendix A, Figure A4). However, it is alternating (not neighbouring) side chains in the primary structure that are in close proximity in the same fringe in the secondary structure—as shown in the schematic in Figure (Figure 5e). This observation is by no means clear from simple inspection of the primary structure of the GXM serotypes.

The side chain fringes are exposed and thus potentially form Ab binding surfaces—this is seen more clearly in the space filling structure representations in the bottom row of Figure 5. The molecular surface differs considerably across the serogroups. The fringes in the cnD structure (Figure 5b) comprise repeated patches of βDGlcA -βDXyl side chain pairs in close proximity, with gaps between them where the mannose backbone is exposed. In contrast, in cnA (Figure 5c) the additional βDXyl(1→2) side chain in each trimer creates a very different binding surface, forming βDGlcA-βDXyl-βDXyl patches in a continuous band in each side chain fringe. Further, in cnA’ (Figure 5d) the 6-O-acetyl substitution forms a highly exposed hydrophobic ridge between the side chain fringes, which further conceals the mannan backbone.

**Figure A2:**
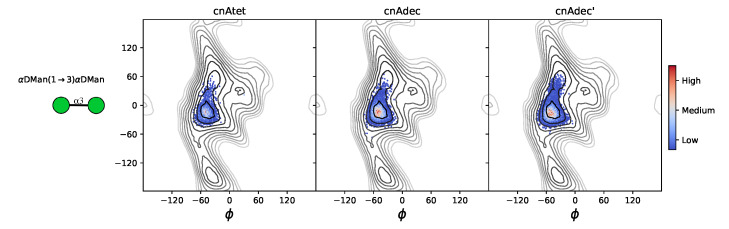
Heat maps of the distribution of the central backbone αDMan(1→3)αDMan glycosidic linkage orientation for a the cnAtet tetrasaccharide fragment (**left**) and decasaccharide cnAdec (**middle**) as well as a 6-O-acetylated decasaccharide cnAdec’ (**right**). Glycosidic linkage orientations are shown as rotations of the linkage dihedral angles ϕ and ψ.

**Figure A3:**
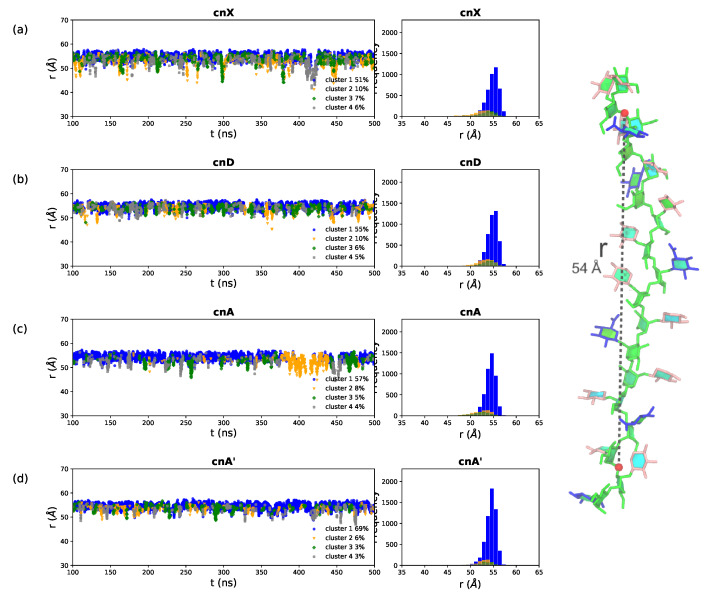
The end-to-end distance, *r*, time series and corresponding histograms of the dominant conformational clusters for the (**a**) cnX, (**b**) cnD, (**c**) cnA and (**d**) cnA’ GXM chains. For each molecule, the end-to-end distance of the dominant conformational cluster is plotted in blue, the secondary conformational cluster in orange, tertiary in green and quaternary in grey. Lesser clusters are not shown. Percentages for each cluster relative to the total simulation are listed in the legends. The first 100 ns of simulation are considered equilibration and are not shown. Here *r* is defined to exclude the two terminal residues on either end of the chain, as the distance from O3 in the second linkage to O3 in the 16th linkage in the 18-mannose backbone—labeled for cnA in the image on the right. The residues and substitutions are coloured as follows: αDMan—green; βDGlcA—blue; βDXyl—pink and 6-OAc—red.

**Figure A4:**
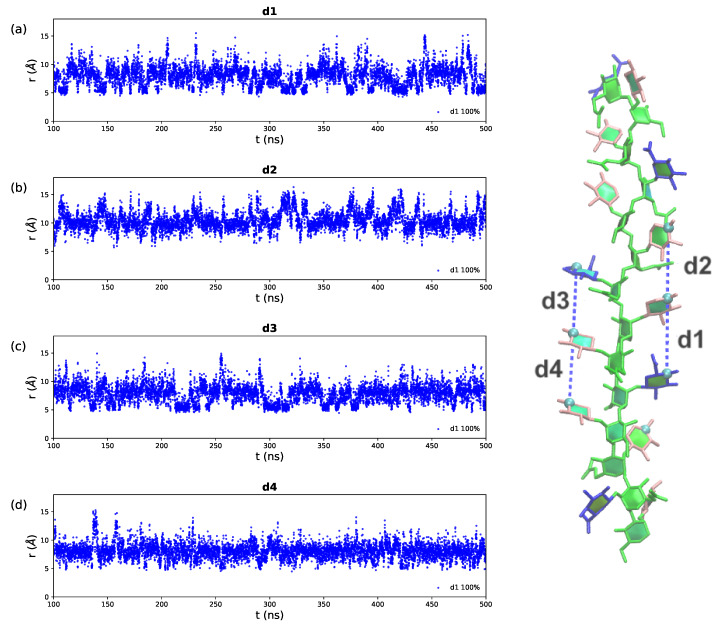
The time series for the distances between interacting side chains in the cnA’ molecule labeled on the right. (**a**) The βDGlcA and βDXyl distance d1, (**b**) the βDXyl and βDXyl distance d2 (**c**) the βDGlcA and βDXyl distance d3 and (**d**) the βDXyl and βDXyl distance d4. Here in each case *d* is defined to as the distance from C4 in one residue to C4 in the proximal side chain residue. The first 100 ns of simulation are considered equilibration and are not shown. The residues and substitutions are coloured as follows: αDMan—green; βDGlcA—blue; βDXyl—pink and 6-OAc—red.

The shorter chain fragments do not differ in conformation from the larger oligosaccharides. For the serogroup A tetrasaccharide cnAtet (Figure 5f) and the two decasaccharides cnAdec and cnAdec’ (Figure 5g), the side chains and the backbone glycosidic linkages have the same orientations as in the 6-RU saccharides (see Appendix A, Figure A2)—there is no conformational change in these shorter strands as compared to the longer chains.

### 2.3. GXM Self-Aggregation

**Figure 6:**
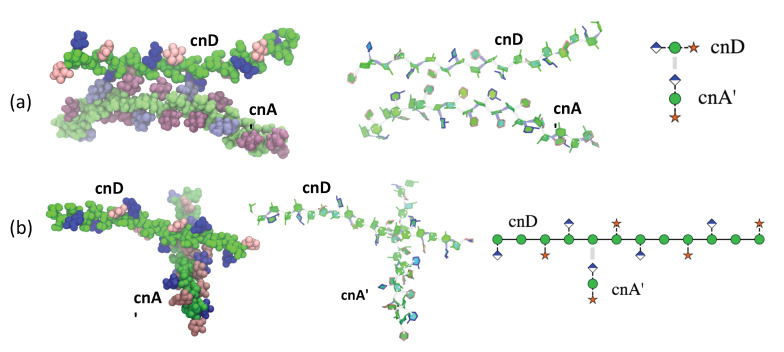
Examples of self-aggregation interactions between 6-RU strands of cnD and cnA in (**a**) parallel and (**b**) orthogonal arrangements. Molecules are depicted using the VMD [26] van der Waals representation (left); and the VMD PaperChain and Twister representations (middle) and a schematic (right). The saccharide residues are coloured as follows: αDMan—green; βDGlcA—blue; βDXyl—pink; and 6-OAc—red.

Physical chemical studies have provided evidence that GXM molecules have the capacity for self-aggregation, but the mechanism for this effect is unknown [22]. Our simulation of cnD and cnA’ together shows evidence for self-aggregation, after 300 ns of equilibration of the system where the chains diffused in the water box. After this, there were repeated instances of prolonged (≈50 ns) spontaneous inter-locking of the two chains. We found two modes of interaction between GXM chains to occur. Firstly, a parallel head-to-tail orientation, where the side chains of one GXM molecule interacted with the backbone mannan of the neighbouring molecule. Figure 6a shows an example of this interaction. Here the cnA’ is rotated around the backbone axis by 90° relative to cnD, to bring the side chains of the cnA’ molecule into contact with the mannan backbone in cnD (illustrated in the cross-sectional schematic on the right of Figure 6a). The interacting side chains in both strands are in the major “face-on” orientation.

Secondly, an orthogonal interaction occurred, which brings the two chains perpendicular to each other. Figure 6b shows an example of this. Here chain latching of cnA’ occurs in a complementary “gap” region of the cnD chain where a βDXyl side chain is missing and the mannose backbone is exposed. This allows the cnA’ side chains to interact directly with multiple hydrogen bonds to the cnD mannan backbone. The flexible side chains on either edge of the gap in cnD orient “face-on” to accommodate the cnA’ chain. This general mode of interaction is illustrated in the schematic on the right of Figure 6b, where the cnA’ chain is drawn in cross-section.

## 3. Discussion

Our simulations reveal the GXM secondary structure to comprise an extended, relatively inflexible, mannan backbone decorated by hydrophyllic fringes of βDGlcA and βDXYl side chains and a hydrophobic ridge of 6-O-acetyl substitutions. A rigid GXM molecule could help explain some experimental observations from physical chemical studies of GXM and cryptococcal capsules. Probing the polysaccharide capsule with polystyrene beads manipulated with optical tweezers to measure its Young’s modulus revealed a relatively stiff structure [28]. The modelling prediction of a linear, inflexible GXM molecule suggests an explanation for the stiffness of the capsule, despite it being composed primarily of water [29]. Further, rigid GXM molecules imply that any increase in molecular mass will translate into molecular length. In this regard, the macroscopic diameter of the capsule size was correlated to the GXM effective diameter measured by light scattering, leading to the proposal that capsule growth is mediated by linear extension of polysaccharide molecules [30]. The prediction that GXM is a rigid molecule is consistent with this observation and suggests how linear growth of individual molecules can translate into increases in capsule size.

Recently, serotype D strains were found to have higher cell surface hydrophobicity than the other serotypes [31]. Although the connection between the predicted molecular structure and cell surface hydrophobicity is not immediately obvious, we note this association and suggest that perhaps the reduced hydrophillic fringes with the concomitant higher exposure of the mannose backbone in cnD could contribute to this effect.

The GXM secondary structures predicted by this work provide a mechanistic rationale for the importance of 6-O-acetylation for antibody binding: the O-acetyl groups line up in a hydrophobic ridge along the mannan backbone—they are highly exposed for antibody binding and their absence in cnA dramatically alters the exposed binding surface. In addition, O-acetylation reduces the flexibility of the mannan backbone, which may further improve antibody binding be providing a more defined epitope. There is a precedent for this in the Vi capsular polysaccharide of *Salmonella typhi* which is a linear homopolymer of →4)αDGalNAc*p*(1→ variably O-acetylated at the C-3 position. Immunity of Vi is closely linked to the degree of O-acetylation, a phenomenon which has been attributed to decreased backbone flexibility [32]; modelling has shown O-acetylation to reduce the molecular flexibility of Vi dramatically [33]. For the GXM mannan backbone, the slight constraint that O-acetylation imposes on every acetylated sugar may be additive in maintaining preferential structures recognised by antibodies.

It may be that protective mAbs in general bind the O-acetyl ridge (which is more conformationally defined) and that the non-protective Abs bind the far more mobile side chain ridges. For example, the synthetic heptasaccharide (corresponding roughly to an M2 triad) recognised by a number of mAbs (all “non-protective”) may induce Ab binding at the side-chain fringe and not the backbone, as the acetylation pattern did not affect binding [21]. Further, Crp127 is an anti-GXM mAb whose binding shows strong serotype dependence, with serotype D strains bound most strongly, followed by serotype A strains, with little interaction with the *C. gattii* serotypes B and C [34]. Following our model, it is likely that the Ab binds primarily to the exposed 6-O-Ac ridge on the backbone, not the fringe regions. The reason for antigenic difference in *C. gatti* is likely the different binding surface exposed: the additional βDXyl(1→4) side chains in serotypes B and C may further hide the mannose backbone and block binding. However, comparative modelling of the C. gattii serotypes B and C, which feature the βDXyl(1→4) side chains not investigated in this work, are necessary to confirm this hypothesis.

The GXM structures also provide a mechanistic rationale for why short GXM fragments do not bind to protective antibodies: they are of insufficient length to reproduce the binding surfaces of the GXM polysaccharide: the tetrasaccharide is too short to reproduce the GXM O-acetyl ridge or side chain fringes, which may explain its lack of success as a vaccine candidate. In contrast, the cnAdec’ 6-O-acetylates decasaccharide produces a representative segment of the side chain fringes and O-acetyl ridge in the cnA’ strand, which explains the success of this epitope in eliciting antibody binding [18]. To reproduce the secondary structure motif in cnA’ thus requires a stretch of at least two triads, not the single triad (pentasaccharide) that is suggested by the primary structure. This work thus illustrates the potential utility of molecular modelling in informing the rational design of conjugate vaccines.

Given the inflexibility of the mannan backbone, the role of GXM in the capsule is likely as a scaffold molecule, giving structure and support to the capsule. In addition, this work provides a mechanism for self-aggregation of GXM chains [22,24]—chain interlocking occurs spontaneously with complementary stretches on neighbouring GXM strands via strong self-association interactions between mobile side chains and the mannan backbone on the neighbouring strand. We identified two modes of interlocking—a parallel orientation and an orthogonal orientation—both of which could be useful in building a capsule. The chemical structure of GXM predicts linear molecules, but physical chemical studies imply that GXM is a branched structure in solution with properties of a dendrimer [35]. The finding that modelling suggests that GXM molecules have the capacity for self-association provides a mechanism for reconciling molecular linearity with solution branching structures without having to invoke the presence of yet undiscovered chemical structures required for making branched molecules. Consequently, it is possible to envisage how self-associations of individual GXM molecules assemble to form dendrimers by non-covalent interactions.

A function of the side chains could be to facilitate interlocking of the GXM strands: flexible side chains can orient to accommodate and enhance the interlocking interaction. Interaction is likely to be further facilitated in the presence of divalent cations [23] which could pull the chains together. However, as divalent cations cannot be accurately modelled with the standard biomolecular force fields, an alternative simulation methodology will be required to investigate this. Further, varying motifs in a GXM chains may facilitate the random inter-locking of GXM strands: backbone regions with fewer side chains (and hence gaps exposing the mannan backbone) are compatible with regions with increased side chains. For example, if M1 motifs interlock with M2 motifs, then chain interlocking would be facilitated by a serotype D chain (primarily M1) with occasional insertion of M2 motifs. This work therefore also provides a rationale for the unusual heterogeneity in the GXM polysaccharide. There is considerable scope for exploring the mechanisms of self-association with simulations of multiple GXM molecules, and the role of the galactomannan polysaccharide in the capsule, in future work.

## 4. Materials and Methods

To model the GXM polysaccharides, we followed our established systematic molecular modelling approach used in previous work [36,37,38,39,40]. First, we determined the low energy conformations of each of the glycosidic linkages in the polysaccharide by calculation of the ϕ, ψ potential of mean force (PMF) for the corresponding disaccharides. We then used these preferred conformations to build reasonable initial conformations for each of the seven oligosaccharides (cnX, cnD, cnA, cnA’, cnAtet, cnAdec and cnAdec’) with our CarbBuilder software [41]. These starting structures were then minimised and molecular dynamics simulations run in a water box (with addition of neutralising counter ions when the system contained the charged βDGlcA residues). The saccharide conformations were then extracted from the simulation trajectories and analysed to establish the dominant conformations and chain dynamics. Details are as follows.

### 4.1. Disaccharide PMF Calculations

The orientations of the glycosidic linkages in the GXM polysaccharides are each described by two dihedral angles, ϕ and ψ. For the backbone αDMan(1→3)αDMan linkages, these are defined here as ϕ= H1–C1–O3’–C3’ and ψ= C1–O3’–C3’–H3’. For both the βDGlcA(1→2)αDMan the βDXyl(1→2)αDMan side chain linkages, ϕ= H1–C1–O2’–C2’ and ψ= C1–O2’–C2’–H2’.

We identified the low-energy conformations of the each of the glycosidic linkages in isolation by calculation of the potential of mean force (PMF) for rotation about the ϕ and ψ dihedral angles in representative disaccharides; viz., αDMan(1→3)αDMan; βDGlc(1→2)αDMan and βDXyl(1→2)αDMan (note that the βDGlcA(1→2)αDMan linkage was approximated with the uncharged βDGlc(1→2)αDMan disaccharide). PMFs were calculated with the metadynamics [42] routine incorporated into NAMD [43] using the glycosidic linkage torsion angles as collective variables and the CHARMM36 additive force field for carbohydrates [44,45]. All PMF surfaces were calculated in gas-phase. Gas phase PMFs for uncharged disaccharides have been demonstrated to be a reasonable approximation to solution PMF in a polysaccharide [37,46,47]. Each standard metadynamics simulation comprised a 1500 ns MD simulation, with a Gaussian hill height of 0.5, hill frequency of 1000 and a hill width of 2.5 degrees. The three PMFs appear in Appendix A, Figure A1.

### 4.2. Molecular Dynamics Simulations

All simulations were performed with the NAMD molecular dynamics program [43] version 2.12 (employing NAMD CUDA extensions for calculation of long-range electrostatics and nonbonded forces on graphics processing units [48]). Carbohydrates were modelled with the CHARMM36 additive force field for carbohydrates [44,45] and water was simulated with the TIP3P model [49]. Initial configurations of seven oligosaccharides (cnX, cnD, cnA, cnA’, cnAtet, cnAdec and cnAdec’) were built using our CarbBuilder software [41,50] v1.2.23 which employs the psfgen tool to create “protein structure” (psf) files for modelling with the CHARMM force field and the NAMD molecular dynamics program. These initial oligosaccharide structures were optimised with 20,000 steps of standard NAMD minimisation in vacuum and then solvated (using the *solvate* plugin to the visual molecular dynamics (VMD) [26] analysis package) in a periodic cubic unit cell. The charged saccharides (cnD, cnA, cnA’, cnAdec and cnAdec’) incorporated randomly distributed sodium ions to electrostatically neutralise the system.

All MD simulations were preceded by a 30,000 step minimisation phase, with a temperature control and equilibration regime involving 10 K temperature reassignments from 10 K culminating in a maximum temperature of 300 K. Equations of motion were integrated using a Leap-Frog Verlet integrator with a step size of 1 fs and periodic boundary conditions. Simulations were performed under isothermal-isobaric (nPT) conditions at 300 K maintained using a Langevin piston barostat [51] and a Nose-Hoover [52,53] thermostat. Long-range electrostatic interactions were treated using particle mesh Ewald (PME) summation, with κ=0.20 Å^−1^ and 1 Å PME grid spacing. Non-bonded interactions were truncated with a switching function applied between 12.0 and 15.0 Å to groups with integer charge. The 1–4 interactions were not scaled, in accordance with the CHARMM force field recommendations. Each system was equilibrated 0.03 ns with a cycled temperature increase from 0 to 300 K in 10 K increments, each cycle commencing with a 10,000 step energy minimisation followed by a 0.001 ns MD simulation at the specified temperature until 300 K. For all simulations, structures were collected at intervals of 250 ps for analysis.

#### 4.2.1. Simulations of Single Saccharide Chains

The 6-RU strands (cnX, cnD, cnA and cnA’) were placed in the centre of a cubic water box with sides of 80 Å, while the tetrasaccharide (cnAtet) and decasaccharide (cnAdec and cnAdec’) strands employed box lengths of 50 Åand 60 /AA, respectively. The charged systems were neutralised with Na^+^ ions: 6 ions for cnD, cnA and cnA’; 1 ion for cnAdec and cnAdec’. All 6-RU MD simulations ran for 500 ns (with the first 100 ns treated as equilibration) and the simulations of the shorter fragments (cnAtet, cnAdec and cnAdec’) ran for 125 ns (with the first 25 ns treated as equilibration).

#### 4.2.2. Simulation of Two Saccharide Chains: cnA’ and cnD

For the combined simulation of cnA’ and cnD, 6-RU strands of each polysaccharide were placed in close proximity within a 80 Å water box with 12 neutralising Na^+^ ions. The combined simulation of cnA’ and cnD ran for 1200 ns, to allow the molecules to relax, diffuse and spontaneously interact.

### 4.3. Data Analysis

Analysis of the simulations used time series frames 25 ps apart. Molecular conformations extracted from the MD simulations were depicted with VMD, where necessary using the PaperChain and Twister visualisation algorithms for carbohydrates [27] to highlight the hexose rings and backbone, respectively. Dihedral angles and end-to-end distances from the simulations were extracted using VMD’s Tcl scripting interface and statistical values calculated with in-house Python scripts. Conformations from all MD simulation trajectories were clustered using VMD’s internal *measure cluster* command. Clustering analysis used time series frames 250 ps apart, discarding the first 100 ns as equilibration. First, all simulation conformations were aligned on the two middle mannose residues in the 18 mannose chain (i.e., the ninth and tenth mannose residues). Then all conformations were clustered into families according to a rmsd fit (cutoff of 5 Å) to all the saccharide rings and glycosidic linkage oxygens in the molecules, ignoring the first two and last two residues in the chain. Only the top four clusters are reported; more minor clusters are ignored.

## Abbreviations

The following abbreviations are used in this manuscript:

Ab	antibody
cnA	6-RU chains of GXM serogroup A primary motif
cnA’	6-RU chains of GXM serogroup A primary motif, 6-*O*-acetylated
	on every second and third mannose in the RU
cnD	6-RU chains of GXM serogroup D primary motif
cnAtet	a tetrasaccharide of GXM serogroup A primary motif
cnAdec	a decasaccharide of GXM serogroup A primary motif
cnAdec’	a decasaccharide of GXM Serogroup A primary motif 6-*O*-acetylated
	on the second and third mannose and last mannose
GXM	glucuronoxylomannan polysaccharide
MD	molecular dynamics
PMF	potential of mean force
RU	repeat unit
